# Analogues of amphibian alkaloids: total synthesis of (5*R*,8*S*,8a*S*)-(−)-8-methyl-5-pentyloctahydroindolizine (8-*epi*-indolizidine 209B) **and** [(1*S*,4*R*,9a*S*)-(−)-4-pentyloctahydro-2*H*-quinolizin-1-yl]methanol

**DOI:** 10.1186/1860-5397-4-5

**Published:** 2008-01-18

**Authors:** Joseph P Michael, Claudia Accone, Charles B de Koning, Christiaan W van der Westhuyzen

**Affiliations:** 1 Molecular Sciences Institute, School of Chemistry, University of the Witwatersrand, PO Wits 2050, South Africa

## Abstract

**Background:**

Prior work from these laboratories has centred on the development of enaminones as versatile intermediates for the synthesis of alkaloids and other nitrogen-containing heterocycles. In this paper we describe the enantioselective synthesis of indolizidine and quinolizidine analogues of bicyclic amphibian alkaloids *via* pyrrolidinylidene- and piperidinylidene-containing enaminones.

**Results:**

Our previously reported synthesis of racemic 8-*epi*-indolizidine 209B has been extended to the laevorotatory enantiomer, (−)**-9**. Attempts to adapt the synthetic route in order to obtain quinolizidine analogues revealed that a key piperidinylidene-containing enaminone intermediate (+)**-28** was less tractable than its pyrrolidinylidene counterpart, thereby necessitating modifications that included timing changes and additional protection–deprotection steps. A successful synthesis of [(1*S*,4*R*,9a*S*)-4-pentyloctahydro-2*H*-quinolizin-1-yl]methanol (−)**-41** from the chiral amine *tert*-butyl (3*R*)-3-{benzyl[(1*R*)-1-phenylethyl]amino}octanoate (+)**-14** was achieved in 14 steps and an overall yield of 20.4%.

**Conclusion:**

The methodology reported in this article was successfully applied to the enantioselective synthesis of the title compounds. It paves the way for the total synthesis of a range of *cis*-5,8-disubstituted indolizidines and *cis*-1,4-disubstituted quinolizidines, as well as the naturally occurring *trans*-disubstituted alkaloids.

## Background

**Figure 1:**
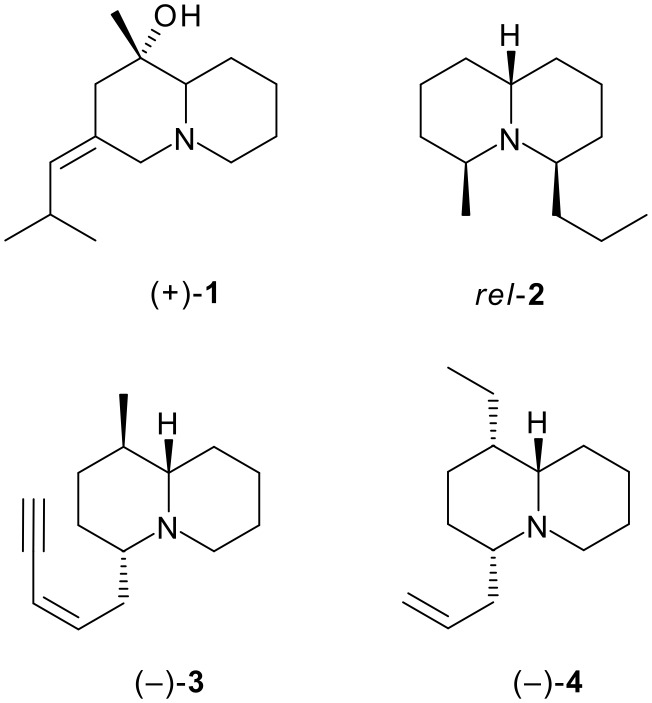
Representative quinolizidine alkaloids from amphibians.

The astonishingly diverse range of alkaloids isolated from the skins of amphibians includes numerous 1-azabicyclic systems belonging to the indolizidine (1-azabicyclo[4.3.0]nonane), quinolizidine (1-azabicyclo[4.4.0]decane) and lehmizidine (1-azabicyclo[5.3.0]decane) classes [1–2]. The first of these classes is by far the most populous, and has commanded enormous attention from organic chemists stimulated by the challenges of designing novel total syntheses [3]. The more recently discovered amphibian quinolizidines constitute a smaller group of alkaloids; they embrace homopumiliotoxins (*e.g.* (+)-homopumiliotoxin 223G **1**; Figure 1) and related systems, 4,6-disubstituted quinolizidines (*e.g. rel*-quinolizidine 195C **2**) and 1,4-disubstituted quinolizidines (*e.g.* (−)-quinolizidine 217A **3**). In the latter group, it appears that most of the well-characterised alkaloids have a 1,4-*trans* disposition of the substituents; the only alkaloid in which the substituents are unambiguously *cis* is (−)-quinolizidine 207I **4**. Comparatively few syntheses of quinolizidine 207I, 217A and related compounds have been reported [4–9].

**Figure 2:**
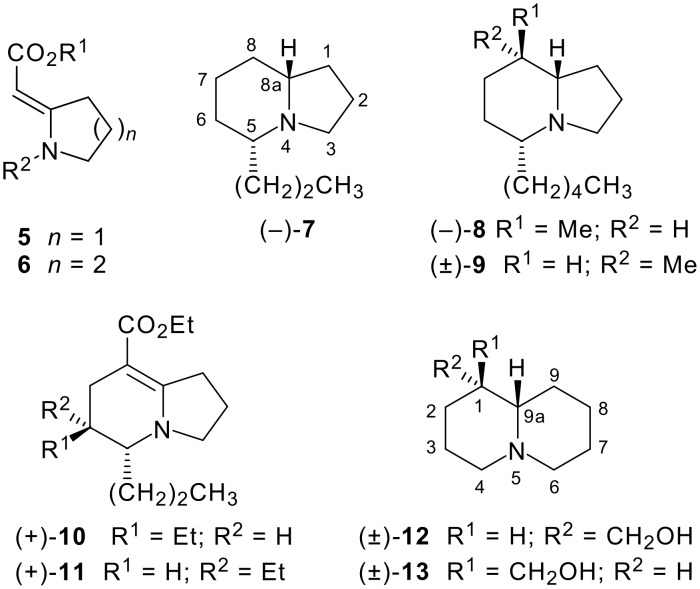
Indolizidines and quinolizidines prepared from enaminone precursors **5** and **6**. The conventional numbering scheme for both bicyclic systems is also shown.

As part of a long-standing investigation into the utility of pyrrolidinylidene- and piperidinylidene-containing enaminones (vinylogous urethanes) **5** and **6** as key intermediates in the synthesis of alkaloids and other nitrogen-containing heterocycles [10], we previously reported total syntheses of (−)-indolizidine 167B **7** [11–12], the 5,8-disubstituted indolizidine (−)-209B **8** and its racemic diastereomer (±)**-9** [13], and the 5,6,8-trisubstituted indolizidines (+)**-10** and (+)**-11** [14], among other similar compounds (Figure 2). While our attempts to prepare quinolizidines have been less successful, we have synthesised two simple lupin alkaloids, lupinine **12** and epilupinine **13**, in racemic form [15]. Although it might seem that reactions of the enaminones **5** and **6** should be directly comparable, we [15–16] and others [17–18] have previously found unexpected differences in the preparation and reactions of cyclic enaminones of different ring sizes. In this article we report our progress in preparing 1,4-disubstituted quinolizidine analogues of amphibian alkaloids by an extension of our approach to the synthesis of 5,8-disubstituted indolizidine alkaloids [19].

## Results and Discussion

**Scheme 1:**
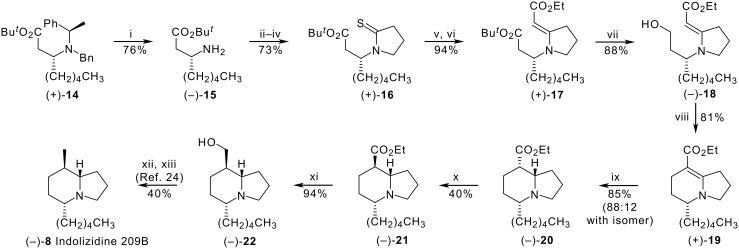
*Reagents:* (i) H_2_ (7 atm), 10% Pd/C, AcOH, rt; (ii) Cl(CH_2_)_3_COCl, NaHCO_3_, CHCl_3_, reflux; (iii) KOBu*^t^*, Bu*^t^*OH, rt; (iv) Lawesson's reagent, PhMe, reflux; (v) BrCH_2_CO_2_Et, MeCN, rt; (vi) Ph_3_P, Et_3_N, MeCN, rt; (vii) LiAlH_4_, THF, rt; (viii) I_2_, imidazole, Ph_3_P, PhMe, 110 °C; (ix) H_2_ (1 atm), PtO_2_, AcOH, rt; (x) NaOEt (cat.), EtOH, reflux; (xi) LiAlH_4_, THF, 0 °C to rt; (xii) MeSO_2_Cl, NEt_3_, CH_2_Cl_2_, 0 °C to rt; (xiii) LiEt_3_BH, THF, 0 °C.

Steps in our reported total synthesis of (−)-indolizidine (−)-209B **8** [13] are shown in Scheme 1. Absolute stereocontrol resulted from use of the Davies protocol [20–21], whereby the homochiral amine (+)**-14** prepared from *tert*-butyl (*E*)-oct-2-enoate and (*R*)-*N*-benzyl-1-phenylethylamine, was converted into the primary amine (−)**-15** and thence in several steps into the thiolactam (+)**-16**. Eschenmoser sulfide contraction [22–23] with ethyl bromoacetate yielded the key enaminone intermediate (+)**-17**, chemoselective reduction of the saturated ester of which produced the alcohol (−)**-18**. The bicyclic core of the alkaloid was then constructed by a cycloalkylation that took advantage of the nucleophilic reactivity of the enaminone, following which a chemoselective and reasonably diastereoselective (88:12) reduction of the alkene bond of the bicyclic enaminone (+)**-19** set up the desired stereochemistry at C-8 and C-8a. Epimerisation of the ester in the reduced compound (−)**-20** produced (−)**-21**, reduction of which gave the alcohol (−)**-22**. Reduction of the corresponding methanesulfonate with lithium triethylborohydride, as described by Holmes *et al*. [24], completed the total synthesis of (−)-indolizidine 209B **8**.

**Scheme 2:**
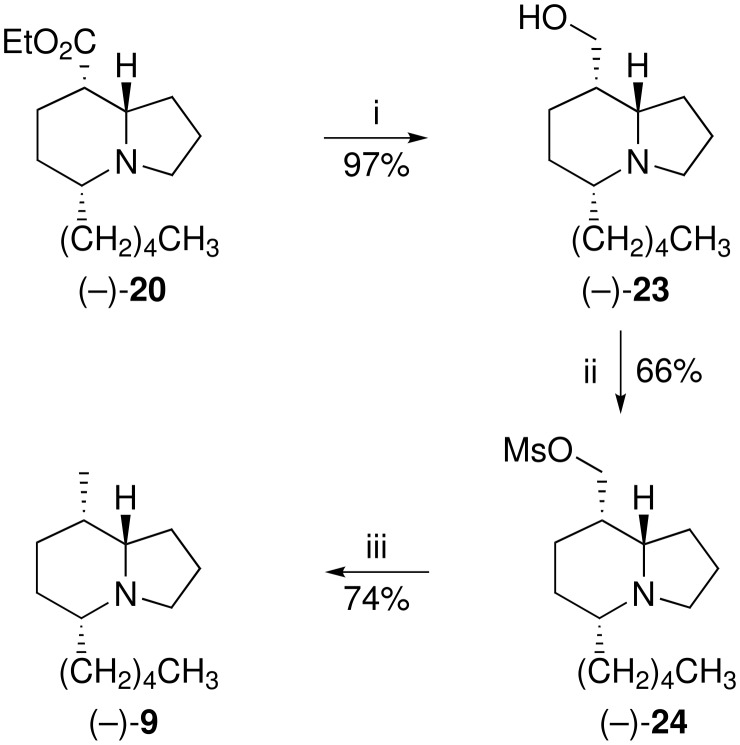
*Reagents:* (i) LiAlH_4_, THF, 0 °C to rt; (ii) MeSO_2_Cl, NEt_3_, CH_2_Cl_2_, 0 °C to rt; (iii) Raney Ni, EtOH, reflux.

As a postscript to the above synthesis, we have now completed an enantioselective synthesis (Scheme 2) of the indolizidine analogue of **8**, *viz*. (5*R*,8*S*,8a*S*)-8-*epi*-indolizidine 209B (−)**-9**, which we had previously made as a racemate [13]. Intermediate (−)**-20** was reduced with lithium aluminium hydride in diethyl ether to give the alcohol (−)**-23** in 97% yield (See Supporting Information File 1 for full experimental data). The corresponding methanesulfonate (−)**-24** (66%) was then defunctionalised by an improved procedure, which entailed treatment with freshly prepared Raney nickel [25] in boiling ethanol to give (−)-(5*R*,8*S*,8a*S*)-8-methyl-5-pentyloctahydroindolizine (8-*epi*-indolizidine 209B) **9** in 74% yield. The spectroscopic data for this product agreed with those reported for the racemate. Support for the *cis*-relationship of the hydrogen atoms at C-5 and C-8a in all of these compounds was provided by Bohlmann bands [26] at *ca*. 2790 cm^−1^ in the FTIR spectra, a feature that also implies a *trans*-disposition of the lone pair and 8a-H across the ring junction.

**Scheme 3:**
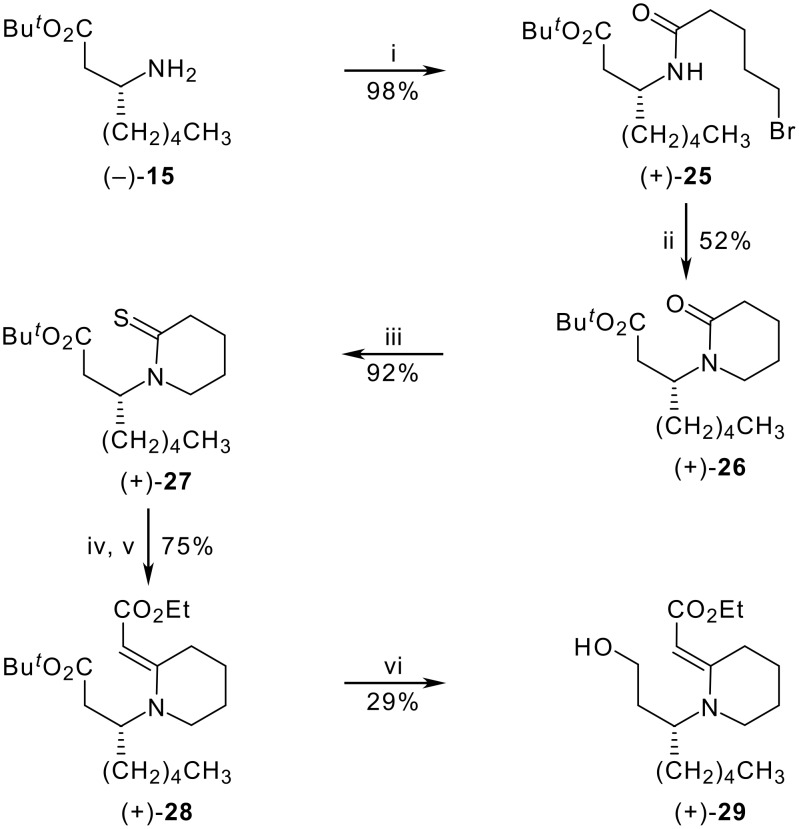
*Reagents:* (i) Br(CH_2_)_4_COCl, NaHCO_3_, ClCH_2_CH_2_Cl, rt; (ii) NaH, Bu_4_NI, DMF, rt; (iii) Lawesson's reagent, PhMe, reflux; (iv) BrCH_2_CO_2_Et, MeCN, rt; (v) P(OEt)_3_, Et_3_N, MeCN, rt; (vi) LiAlH_4_, THF, rt.

Extending the route illustrated in Scheme 1 to the synthesis of quinolizidine analogues required initial acylation of the chiral amine (−)**-15**, prepared as described in our prior work [13], with 5-bromopentanoyl chloride (obtained in two steps from δ-valerolactone) [27–28]. This afforded *tert*-butyl (3*R*)-[(5-bromopentanoyl)amino]octanoate (+)-**25** in 98% yield (Scheme 3). However, subsequent cyclisation to the lactam (+)**-26** was troublesome, giving at best a yield of 52% when performed with sodium hydride and tetrabutylammonium iodide in *N*,*N*-dimethylformamide. An effortless thionation of **26** with Lawesson's reagent in boiling toluene produced the thiolactam (+)**-27** in 92% yield. Eschenmoser sulfide contraction was then effected by first treating the thiolactam with ethyl bromoacetate, after which reaction of the resulting S-alkylated intermediate with triethyl phosphite and triethylamine in acetonitrile gave the vinylogous urethane (+)**-28** in 75% yield.

At this stage, however, our fears of the discrepant behaviour of five- and six-membered enaminones proved to be all too well founded. In the indolizidine series, the robust enaminone **17** survived reduction with lithium aluminium hydride, leaving only the saturated ester to be reduced. With the six-membered analogue **28**, the enaminone unit was far more susceptible to reduction, and despite many attempts to modify conditions, over-reduction led to a plethora of basic products that could neither be separated nor properly characterised. Although the desired alcohol (+)**-29** containing an intact enaminone system could be isolated on occasion, the best yield obtained was 29% when the reaction was not allowed to go to completion. Thus a change of strategy was required to produce **29**, the pivotal intermediate from which the quinolizidine nucleus needs to be constructed.

**Scheme 4:**
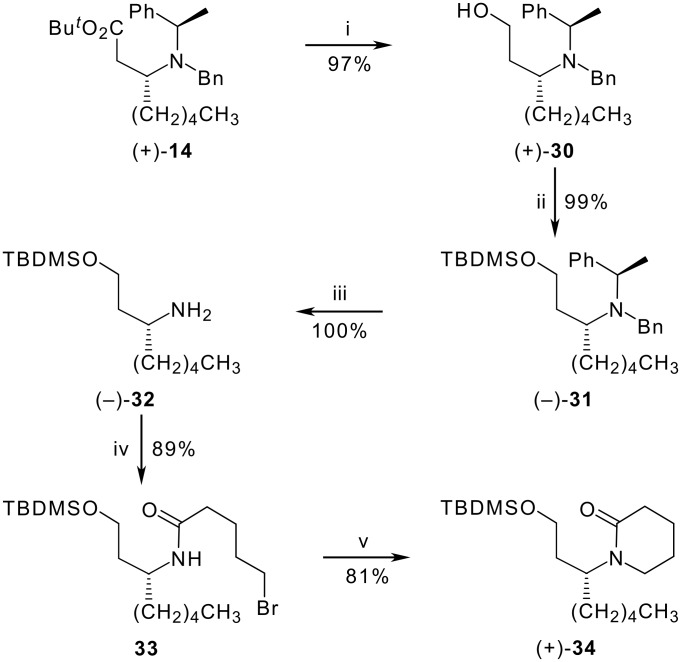
*Reagents:* (i) LiAlH_4_, Et_2_O, 0 °C, then add (+)-**2** in Et_2_O, rt; (ii) TBDMSCl, imidazole, DMF, rt; (iii) H_2_ (5 atm), 20% Pd(OH)_2_/C, AcOH, rt; (iv) Br(CH_2_)_4_COCl, NaHCO_3_, ClCH_2_CH_2_Cl, rt; (v) *t*-BuOK, THF, rt, 25 min.

The reduction of the *tert*-butyl ester clearly needed to be performed at an early stage of the synthesis before the introduction of other incompatible functional groups (lactam, thiolactam, enaminone). The only feasible option was to go back to the chiral amine (+)**-14**, reduction of which with lithium aluminium hydride gave the unstable amino alcohol (+)**-30** in 97% yield as long as the amine was added slowly to a stirred suspension of the hydride in diethyl ether (Scheme 4). If the order of addition were reversed, the best yield obtained was 48%. The amino alcohol was protected as its *tert*-butyl(dimethyl)silyl ether (−)**-31** (99%) before hydrogenolysis of the benzyl groups over Pearlman's catalyst in glacial acetic acid gave the free amine (−)**-32** in quantitative yield. Treatment with 5-bromopentanoyl chloride as described above afforded the unstable bromoamide **33** as an orange oil in 89% yield. In this case, cyclisation of the crude intermediate to the lactam (+)**-34** was most successfully effected by adding potassium *tert*-butoxide to a solution of the bromoamide in dry tetrahydrofuran at room temperature, a yield of 81% being obtained by keeping the reaction time short (25 min). To our dismay, however, the attempted thionation of **34** with Lawesson's reagent under a variety of conditions was uniformly unsuccessful, apparently because the silyl ether failed to survive the reaction conditions.

**Scheme 5:**
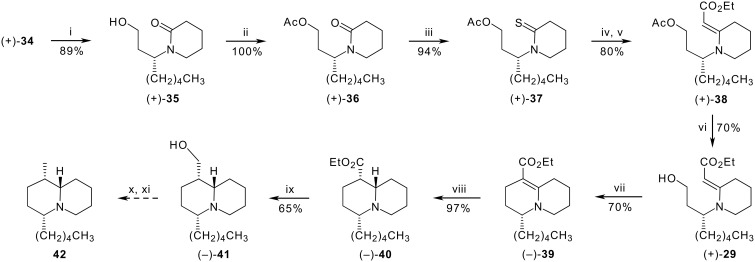
*Reagents:* (i) aq. HF (40%), MeOH, rt; (ii) Ac_2_O, pyridine, 0 °C to rt; (iii) Lawesson's reagent, PhMe, reflux; (iv) BrCH_2_CO_2_Et, MeCN, rt; (v) Ph_3_P, Et_3_N, MeCN, rt; (vi) K_2_CO_3_, MeOH, rt; (vii) I_2_, PPh_3_, imidazole, MeCN-PhMe (2:1), reflux; (viii) H_2_ (1 atm), PtO_2_, AcOH, rt; (ix) LiAlH_4_, THF, 0 °C to rt; (x) MeSO_2_Cl, NEt_3_, CH_2_Cl_2_, 0 °C to rt; (xi) Raney Ni, EtOH, reflux.

Inelegant though it was, we were forced at this stage to change protecting groups on the alcohol. Fortunately, the drop in yield was not too serious when desilylation of **34** with aqueous hydrofluoric acid to give the free alcohol (+)**-35** was followed by acetylation with acetic anhydride in pyridine (Scheme 5). The lactam (+)**-36**, obtained in an overall yield of 89%, was then successfully thionated with Lawesson's reagent in boiling toluene to give the thiolactam (+)**-37** in 94% yield. Finally, reaction with ethyl bromoacetate followed by treatment with triphenylphosphine and triethylamine in acetonitrile give the vinylogous urethane (+)**-38** in 80% yield. Hydrolysis of the acetate with potassium carbonate in methanol then afforded the pivotal alcohol (+)**-29** (70%). The scene was now set for cyclisation to the quinolizidine system. Immediate conversion of the unstable free alcohol into the corresponding iodide with iodine, triphenylphosphine and imidazole in a mixture of toluene and acetonitrile [29] and heating the reaction mixture under reflux gave the desired 3,4,6,7,8,9-hexahydro-2*H*-quinolizine-1-carboxylate (−)**-39** in 70% yield.

In order to introduce the remaining stereogenic centres of the target system, the alkene bond of the bicyclic vinylogous urethane **39** needs to be reduced stereoselectively. Based on our previous success with the indolizidine analogue **19**, we opted for catalytic hydrogenation, which is expected to produce not only a *cis*-relationship between C-1 and C-9a, but also a *cis*-relationship between C-4 and C-9a. The developing chair conformation of the six-membered ring in the transition state should result in an equatorial preference for the pentyl side chain, which in turn should bias the approach of the reductant towards the more remote face of the double bond. Gratifyingly, hydrogenation of intermediate **39** over platinum oxide catalyst in ethanol at a pressure of five atmospheres produced the quinolizidine (−)**-40** as a single diastereomer in 97% yield. The diastereoselectivity is manifestly better than in the indolizidine case. Support for the *cis*-relationship of the hydrogen atoms at positions C-4 and C-9a and the *trans*-ring junction in the product was once again provided by Bohlmann bands in the FTIR spectrum at *ca*. 2790 cm^−1^. However, further confirmation of the relative stereochemistry by consideration of the ^1^H NMR spectrum was not feasible because overlap of signals prevented the extraction of coupling constants for 1-H and 9a-H.

Finally, reduction of the ester to the primary alcohol (−)**-41** was accomplished in moderate yield (65%) with lithium aluminium hydride. Again, coupling constants could not be determined for 1-H and 9a-H. In this case, however, there is good precedent for assigning the relative stereochemistry of the hydroxymethyl substituent at C-1 on the basis of ^13^C chemical shifts. For example, the chemical shift of C-1 in lupinine **12**, which possesses an axial hydroxymethyl substituent, is 38.8 ppm; whereas the corresponding chemical shift in epilupinine **13**, the equatorial hydroxymethyl epimer, is 43.8 ppm [30]. The chemical shift difference of about 5 ppm between the C-1 equatorial and axial hydroxymethyl epimers appears to be general for quinolizidines [31]. A similar effect has been reported for 8-hydroxymethylindolizidine epimers, for which the chemical shift difference is even larger (*ca* 10 ppm) [24]. In the present case, the observed chemical shift of 38.4 ppm for **41** is consistent with an axial disposition of the C-1 substituent, and thus with the expected *cis*-hydrogenation of **39**.

While it would have been desirable to conclude this investigation by preparing (1*S*,4*R*,9a*S*)-4-pentyloctahydro-2*H*-quinolizine **42**, the ring homologue of 8-*epi*-indolizidine 209B, this target eluded us. Attempts to reduce the corresponding methanesulfonate of **41** with Raney nickel in boiling ethanol gave ambiguous results no matter how we modified the reaction conditions.

## Conclusion

Few approaches to 1,4-*cis*-disubstituted quinolizidines and 5,8-*cis*-disubstituted indolizidines of amphibian origin have been reported in the literature. Because the route we have devised proceeds through bicyclic enaminone intermediates in which the alkene bond is located between the bridgehead position and the adjacent site, we have a convenient and dependable method for introducing the correct relative stereochemistry at these two sites by means of catalytic hydrogenation. However, the differences in behaviour of pyrrolidinylidene- and piperidinylidene-containing enaminones that we have come to expect [15–16] was again apparent, necessitating several protection-deprotection steps that lengthened the route to the quinolizidine system. Nevertheless, our success in preparing the chiral alcohol **41** opens up a route to quinolizidine alkaloids containing C-1 methyl substituents (provided, of course, that we can find a better method for deoxygenation, probably by radical-mediated reaction). In addition, alkyl homologues at C-1 should be accessible; one could, for example, replace the alcohol by a leaving group that can be displaced by organometallic reagents (*e.g.* cuprates) of appropriate chain length. Substituents at C-4 can also be varied by choosing appropriate analogues of the chiral amine **14**, which should also be available in both enantiomeric forms by the Davies procedure [32]. Finally, since the pendent substituents in the indolizidine series can be induced to adopt a *trans*-orientation by base-catalysed epimerisation of a carbonyl substituent adjacent to the bridgehead position (*cf* Scheme 1), it should in principle be possible to effect a similar epimerisation in the quinolizidine series, thereby providing a route to most of the known 1,4-disubstituted amphibian quinolizidine alkaloids.

## Supporting Information

File 1 Analogues of amphibian alkaloids - Full experimental details. The Supporting Information File contains detailed experimental procedures and full characterisation data for all new compounds prepared during the synthesis of the two title compounds.
