# Supplementary material for: Analogues of amphibian alkaloids: total synthesis of (5R,8S,8aS)-(−)-8-methyl-5-pentyloctahydroindolizine (8-epi-indolizidine 209B) and [(1S,4R,9aS)-(−)-4-pentyloctahydro-2H-quinolizin-1-yl]methanol
Source: Beilstein J Org Chem. 2008 Jan 18;4:5. doi: 10.1186/1860-5397-4-5 (PMC2241605; doi:10.1186/1860-5397-4-5)
Supplement: File 1 — Analogues of amphibian alkaloids - Full experimental details. The Supporting Information File contains detailed experimental procedures and full characterisation data for all new compounds prepared during the synthesis of the two title compounds. [file Beilstein_J_Org_Chem-04-05-s001.doc]

**Additional File 1**

**Analogues of amphibian alkaloids: total synthesis of (5*R*,8*S*,8a*S*)-(–)-8-methyl-5-pentyloctahydroindolizine (8-*epi*-indolizidine 209B) and (1*S*,4*R*,9a*S*)-(–)-4-pentyloctahydro-2*H*-quinolizin-1-yl]methanol:**

**Full experimental details**

Joseph P Michael,* Claudia Accone, Charles B de Koning and Christiaan W van der Westhuyzen

**General**

Tetrahydrofuran (THF) was distilled from Na/benzophenone; acetonitrile, *N,N*-dimethylformamide (DMF), dichloromethane, 1,2-dichloroethane and triethylamine from CaH_2_; pyridine from potassium hydroxide; and toluene from Na metal. Commercially available chemicals were used as received. Melting points, recorded on a Reichert hot-stage microscope apparatus, are uncorrected. TLC was performed on aluminium-backed Alugram Sil G/UV_254_ plates pre-coated with 0.25 mm silica gel 60. Column chromatography was carried out on silica gel 60, particle size 0.063–0.200 mm (conventional columns) or Whatman Partisil Prep 40, particle size 0.040–0.063 mm (flash columns). FTIR spectra were recorded on a Bruker Vector 22 spectrometer. NMR spectra were recorded on Bruker AC-200 (200.13 MHz for ^1^H, 50.32 MHz for ^13^C) or 300 (300.139 MHz for ^1^H, 75.035 MHz for ^13^C) spectrometers. CDCl_3_ was used as solvent and TMS as internal standard. DEPT and CH-correlated spectra were routinely used for assignment of signals. *J* values are given in Hz. High-resolution mass spectra were recorded at 70 eV on a VG 70 SEQ mass spectrometer with a MASPEC II data system. Optical rotations were measured on a Jasco DIP-370 polarimeter; [α]_D_ values are given in 10^–1^ deg cm^2^ g^–1^.

**[(5*R*,8*S*,8a*S*)-5-Pentyloctahydroindolizin-8-yl]methanol 23**

LiAlH_4_ (41 mg, 0.88 mmol) and (–)-ethyl (5*R*)-5-pentyloctahydroindolizine-8-carboxylate **20** [1] (236 mg, 0.884 mmol) were stirred together in dried THF (9 cm^3^) under a nitrogen atmosphere at 0 °C for 30 min. The solution was then allowed to warm to room temperature and left for a further 18 h. The reaction was quenched by the sequential addition of H_2_O (0.04 cm^3^), aqueous NaOH solution (15% w/v, 0.04 cm^3^), and finally H_2_O (0.12 cm^3^). After 15 min, the solids were removed by filtration through celite, washed with copious quantities of acetone and then concentrated *in vacuo* to yield an orange oil. Purification by column chromatography (MeOH–acetone 1:4) afforded *[(5*R*,8*S*,8a*S*)-5-pentyloctahydroindolizin-8-yl]methanol* **23** as a pale orange oil (183 mg, 97%); *R_f_* 0.23 (MeOH–acetone 1:4); [α]_D_^19^ –65.1 (*c* 0.76, MeOH) {lit.,[2] –56.3 (*c* 0.67, MeOH)}; *ν*_max_(film/cm^–1^) 3383 (br s, OH), 2928 (S), 2862 (s), 2790 (m, Bohlmann band) and 1046 (m); δ_H_ (200 MHz, CDCl_3_) 5.10–4.40 (1H, br s, O*H*), 4.21 (1H, dd with further fine coupling, *J* = 10.8 and 3.9, C*H*_a_H_b_OH), 3.72 (1H, d, *J* = 10.7, CH_a_*H*_b_OH), 3.30–3.10 (1H, br dt, *J = ca* 5.7 and 3.5, 3-H_eq_), 2.36 (1H, br tdd, *J= ca* 7.0, 3.7 and 2.8, 8a-H), 2.05–1.35 (11H, m), 1.30–1.05 (8H, m) and 0.88 (3H, t, *J* = 6.5, C_4_H_8_*Me*); δ_C_ (50 MHz, CDCl_3_) 67.2, 65.5, 63.9, 51.7, 34.8, 34.5, 32.2, 26.2, 22.5, 31.2, 28.1, 24.2, 20.6 and 14.0. Spectroscopic data agreed with those reported for racemic **23** [1] and for the (–)-enantiomer prepared by Holmes *et al*.[2]

**[(5*R*,8*S*,8a*S*)-5-Pentyloctahydroindolizin-8-yl]methyl methanesulfonate 24**

[(5*R*,8*S*,8a*S*)-5-Pentyloctahydroindolizin-8-yl]methanol **23** (92 mg, 0.43 mmol), methanesulfonyl chloride (0.083 cm^3^, 1.1 mmol) and Et_3_N (0.24 cm^3^, 1.7 mmol) were stirred in dry CH_2_Cl_2_ (7 cm^3^) under a nitrogen atmosphere at 0 °C for 2 h. The solution was poured into saturated aqueous NaHCO_3_ solution (20 cm^3^) and the aqueous layer was extracted with copious quantities of CH_2_Cl_2_. The combined organic layers were dried (MgSO_4_), filtered and concentrated *in vacuo* to yield the crude mesylate as an orange oil. Purification by column chromatography using EtOAc–hexanes (1:2) as eluent afforded *[(5*R*,8*S*,8a*S*)-5-pentyloctahydroindolizin-8-yl]methyl methanesulfonate* **24** as a pale yellow oil (82 mg, 66%); *R_f_* 0.11 (EtOAc–hexane 1:2); [α]_D_^19^ –32.1 (*c* 1.04, absolute EtOH); *ν*_max_(film/cm^–1^) 2954 (s), 2925 (s), 2855 (s), 1361 (m) and 1175 (m); δ_H_ (200 MHz, CDCl_3_) 4.45 and 4.36 (2H, 2 × dd, *J* = 9.7 and 4.9, and *J* = 9.7 and 8.4, C*H*_2_OMs), 3.25–3.13 (1H, m, 3-H_eq_), 3.00 (3H, s, SO_2_*Me*), 2.27–2.10 (2H, m, 5-H and 8a-H), 2.00–1.80, 1.78–1.47 and 1.45–1.10 (18H, clusters of m, C*H* and CH_2_), and 0.89 (3H, t, *J* = 6.5, C_4_H_8_*Me*); δ_C_ (50 MHz, CDCl_3_) 69.4, 65.7, 64.4, 51.9, 37.1, 35.3, 34.5, 32.3, 24.8, and 22.6, 26.8, 26.6, 25.9, 20.5 and 14.0. Spectroscopic data agreed with those reported for racemic **23**.[1]

**(5*R*,8*S*,8a*S*)-8-Methyl-5-pentyloctahydroindolizine (8-*epi*-Indolizidine 209B) 9**

Freshly prepared Raney nickel [3] (*ca* 250 mg, suspension in absolute EtOH, W-2 activity) was added to [(5*R*,8*S*,8a*S*)-5-pentyloctahydroindolizin-8-yl]methyl methanesulfonate **24** (79 mg, 0.26 mmol) in absolute EtOH (10 cm^3^). The mixture was heated under reflux for 18 h under an atmosphere of nitrogen, cooled to room temperature, allowed to settle in a magnetic field to coagulate the metal, after which the supernatant liquid was decanted. Solids were washed with further absolute EtOH, and decanted as before. The combined organic phases were concentrated *in vacuo* using a water bath heated to ≤ 30 °C, to yield an orange oil. Flash chromatography (conc. aq. NH_4_OH–EtOAc 1:49) gave *(5*R*,8*S*,8a*S*)-8-methyl-5-pentyloctahydroindolizine* **9** as a pale yellow oil (41 mg, 74%); *R_f_* 0.28 (MeOH–EtOAc 1:9); [α]_D_^18^ –80.9 (*c* 1.27, absolute EtOH); *ν*_max_(vapour/cm^–1^) 2966 (s), 2935 (s), 2880 (s), 2786 (m, Bohlmann band); δ_H_ (200 MHz, CDCl_3_) 3.25 (1H, br dt, *J = ca* 5.5 and 3.9, 3-H_eq_), 2.30–1.95, 1.95–1.72, 1.70–1.50, 1.50–1.40 and 1.35–1.10 (20H, clusters of m, C*H* and C*H*_2_), 0.95 (3H, d, *J* = 7.0, CH*Me*) and 0.88 (3H, t, *J* = 6.6, C_4_H_8_*Me*); δ_C_ (50 MHz, CDCl_3_) 67.7, 65.1, 52.3, 34.6, 32.3, 25.3, 22.6, 32.0, 29.5, 26.8, 25.8, 20.3, 14.0 and 12.2. Spectroscopic data agreed with those reported for racemic **23**.[1]

***tert*-Butyl (3*R*)-[(5-bromopentanoyl)amino]octanoate 25**

5-Bromopentanoyl chloride [4] (0.56 g, 2.79 mmol) was added to a stirred solution of *tert*-butyl (3*R*)-3-aminooctanoate **15** [1] (0.50 g, 2.32 mmol) in dry dichloroethane (10 cm^3^) containing anhydrous NaHCO_3_ (0.23 g, 2.79 mmol). The reaction mixture was left to stir for 16 h under a nitrogen atmosphere at room temperature. The resulting orange solution was filtered through a pad of celite, which was then washed with CH_2_Cl_2_. The combined filtrate and washings were evaporated *in vacuo* to yield an orange oil (1.06 g), which was purified by column chromatography on silica gel with EtOAc-hexane (7:3) as eluent to give *tert-butyl (3*R*)-[(5-bromopentanoyl)amino]octanoate* **25** as a yellow oil (0.86 g, 98%); R*_f_* 0.68 (EtOAc-hexane 1:1); [α]_D_^22^ + 5.2 (*c* 1.72, absolute EtOH); *v*_max_ (film/cm^−1^) 3294 (w, br, N–H), 2959 (s), 2932 (s), 2861 (m), 1819 (m), 1729 (s, C=O), 1645 (m, C=O), 1546 (m), 1457 (m), 1254 (m), 1158 (m) and 1045 (m); δ_H_ (300 MHz, CDCl_3_) 6.13 (1H, br d, *J* 9.0, N*H*), 4.22-4.16 (1H, m, C*H*N), 3.41 (2H, t, *J* 6.6, C*H*_2_Br), 2.46 (1H, dd, *J* = 15.5 and 5.0, C*H*_a_H_b_CO_2_R), 2.40 (1H, dd, *J* = 15.5 and 5.2, CH_a_*H*_b_CO_2_R), 2.20 (2H, t, *J* = 7.3, C*H*_2_CONH), 1.92–1.87 (2H, m), 1.82–1.76 (2H, m), 1.45 (9H, s, C*Me*_3_), 1.41–1.21 (8H, m) and 0.88 (3H, br t, *J* = 6.7, C_4_H_8_*Me*); δ_C_ (75 MHz, CDCl_3_) 171.5, 171.4, 81.1, 46.1, 39.5, 35.8, 34.1, 33.1, 32.1, 31.5, 28.1, 25.8, 24.2, 22.5 and 13.9; *m/z* (EI) 379 (0.2%, ^81^Br-M^+^), 377 (0.2%, ^79^Br-M^+^), 323 (8, ^81^Br-M^+^ − isobutene), 321 (8, ^79^Br-M^+^ − isobutene), 306 (9), 304 (6), 277 (3), 262 (6), 252 (16), 250 (16), 242 (14), 206 (7), 158 (46), 101 (12), 100 (72), 88 (100), 70 (10), 57 (52) and 55 (33) (Found: M^+^, 377.1577 C_17_H_32_NO_3_^79^Br requires 377.1565. Found: M^+^ – isobutene, 321.0941 C_13_H_24_NO_3_^79^Br requires 321.0940).

***tert*-Butyl (3*R*)-3-(2-oxo-1-piperidinyl)octanoate 26**

Sodium hydride (60% in oil, 27 mg, 1.12 mmol) was added to a stirred solution of *tert*-butyl (3*R*)-[(5-bromopentanoyl)amino]octanoate **25** (113 mg, 0.298 mmol) and tetra-*n*-butylammonium iodide (9 mg, 0.024 mmol, 8 mol%) in dry DMF (2 cm^3^), and the reaction mixture was left to stir at room temperature for 16 h prior to quenching by the slow addition of water (5 cm^3^). The aqueous residue was extracted with diethyl ether (4 × 10 cm^3^). The ethereal extracts were dried (MgSO_4_), filtered and evaporated *in vacuo* to yield a pale yellow oil (62 mg), which was purified by column chromatography on silica gel with EtOAc-hexane (7:3) as eluent to give *tert-butyl (3*R*)-3-(2-oxo-1-piperidinyl)octanoate* **26** as a pale yellow oil (46 mg, 52%); R*_f_* 0.30 (EtOAc-hexane 1:1); [α]_D_^25^ +10.5 (*c* 1.23, absolute EtOH); *v*_max_(film/cm^−1^) 2932 (br s), 2861 (m), 1727 (s, C=O), 1644 (s, C=O), 1487 (m), 1464 (m), 1367 (m), 1348 (m), 1296 (m) and 1166 (s); δ_H_ (300 MHz, CDCl_3_) 4.94–4.84 (1H, br m, C*H*N), 3.22–3.16 and 3.16–3.10 (2H, 2 × m, C*H*_2_N), 2.46 and 2.41–2.36 (4H, overlapping dd *J* = 14.4 and 9.4, C*H*_a_*H*_b_CO_2_R and m, C*H*_2_CON), 1.80–1.73 (4H, m), 1.42 (9H, s, C*Me*_3_), 1.29–1.20 (8H, m) and 0.87 (3H, t, *J* = 6.8, C_4_H_8_*Me*); δ_C_ (75 MHz, CDCl_3_) 170.6, 169.7, 80.5, 50.8, 42.3, 39.0, 32.6, 31.7, 31.6, 27.9, 25.8, 23.2, 22.4, 20.9 and 13.9; *m/z* (EI) 297 (5%, M^+^), 270 (2), 242 (12), 241 (59, M^+^ – isobutene), 240 (23), 224 (51), 199 (11), 196 (15), 184 (33), 182 (76), 171 (26), 170 (48), 168 (11), 152 (18), 142 (12), 126 (38), 125 (19), 100 (86), 99 (20), 98 (35), 88 (14), 84 (17), 82 (15), 57 (100) and 55 (34) (Found: M^+^, 297.2311. C_17_H_31_NO_3_ requires 297.2304).

***tert-*Butyl (3*R*)-3-(2-thioxo-1-piperidinyl)octanoate 27**

A solution of *tert*-butyl (3*R*)-3-(2-oxo-1-piperidinyl)octanoate **26** (0.92 g, 3.08 mmol) and Lawesson’s reagent (0.62 g, 1.54 mmol, 0.5 equiv.) in dry toluene (12 cm^3^) was heated under reflux for 4 h, resulting in an orange solution. The solvent was then evaporated *in vacuo* to yield a dark yellow oil, which was purified by column chromatography on silica gel with CH_2_Cl_2_-hexane (4:1) as eluent to give *tert-butyl (3*R*)-3-(2-thioxo-1-piperidinyl)octanoate* **27** as a yellow oil (0.89 g, 92%); R*_f_* 0.51 (hexane-EtOAc 4:1); [α]_D_^22^ + 19.3 (*c* 1.47, absolute EtOH); *v*_max_(film/cm^−1^) 2955 (s), 2931 (s), 2860 (m), 1725 (s, C=O), 1493 (m), 1442 (m), 1367 (m, C=S), 1350 (m), 1304 (m), 1257 (m), 1153 (s) and 1097 (m); δ_H_ (300 MHz, CDCl_3_) 6.35–6.15 (1H, m, C*H*N), 3.34 (1H, ddd, *J* = 12.8, 7.3 and 5.4, C*H*_a_H_b_N), 3.26 (1H, br dt, *J = ca* 12.8, 6.3, CH_a_*H*_b_N), 3.04 and 2.98 (2H, 2 × dt, *J* = 17.6 and 6.8, C*H*_a_*H*_b_C=S), 2.50 and 2.54–2.44 (2H, overlapping dd *J* = 14.2 and 6.1, and m, C*H*_a_*H*_b_CO_2_R), 1.93–1.81 (2H, m), 1.77–1.71 (2H, m), 1.64–1.53 (1H, m), 1.44 (9H, s, C*Me*_3_), 1.38-1.22 (7H, m) and 0.88 (3H, t, *J* = 7.0, C_4_H_8_*Me*); δ_C_ (75 MHz, CDCl_3_) 201.8, 169.8, 81.2, 57.0, 43.3, 42.3, 38.8, 32.0, 31.7, 28.0, 25.6, 22.4 (2 overlapping signals), 19.8 and 14.0; *m/z* (EI) 313 (21%, M^+^), 256 (100,), 240 (20), 224 (45), 212 (15), 186 (13), 116 (55), 115 (41), 114 (10), 82 (17), 57 (20) and 55 (16^+^) (Found: M^+^, 313.2079. C_17_H_31_NO_2_S requires 313.2076).

***tert*-Butyl (3*R*)-3-[(2*E*)-2-(2-ethoxy-2-oxoethylidene)piperidinyl]octanoate 28**

Ethyl bromoacetate (0.08 cm^3^, 0.12 g, 0.71 mmol) was added dropwise to a stirred solution of *tert*-butyl (3*R*)-3-(2-thioxo-1-piperidinyl)octanoate **27** (0.11 g, 0.35 mmol) in dry MeCN (2 cm^3^). To ensure complete salt formation, the solution was left to stir for 16 h at room temperature under a nitrogen atmosphere. A solution of triethyl phosphite (0.07 cm^3^, 64.1 mg, 0.39 mmol) and triethylamine (0.05 cm^3^, 39.0 mg, 0.39 mmol) in dry MeCN (2 cm^3^) was added to the stirring salt solution, giving a green solution that was left to stir at room temperature for 24 h. H_2_O (15 cm^3^) was added to the reaction mixture, which was extracted with CH_2_Cl_2_ (3 × 10 cm^3^). The combined extracts were dried (MgSO_4_), filtered and evaporated *in vacuo* to yield a brown oil (0.51 g), which was purified by column chromatography on silica gel with hexane-EtOAc (4:1) as eluent to give *tert-butyl (3*R*)-3-[(2E)-2-(2-ethoxy-2-oxoethylidene)piperidinyl]octanoate* **28** as a pale yellow oil (96 mg, 75%); R*_f_* 0.73 (hexane-EtOAc 7:3); [α]_D_^24^ +10.6 (*c* 1.20, absolute EtOH); *v*_max_(film/cm^−1^) 2932 (s), 2870 (m), 1729 (s, C=O), 1682 (m, C=C), 1568 (m), 1458 (m), 1368 (m), 1287 (m), 1156 (s) and 1036 (m); δ_H_ (300 MHz, CDCl_3_) 4.84 (1H, s, =C*H*), 4.23 (1H, quintet, *J* = 7.1, C*H*N), 4.05 (2H, overlapping q, *J* = 7.0, OC*H*_2_Me), 3.20 and 3.09 (2H, 2 × dt, *J* = 11.0 and 6.7, NC*H*_a_*H*_b_), 3.06 (2H, t, *J* = 7.2, C*H*_2_C=), 2.40 (2H, d, *J* = 7.2, C*H*_2_CO_2_R), 1.72 (2H, quintet, *J* = 6.1, C*H*_2_), 1.67–1.60 (2H, m, C*H*_2_Bu), 1.43 (9H, s, C*Me*_3_), 1.40–1.20 and 1.23 (11H, overlapping m and t, *J* = 7.0, 4 × C*H*_2_ and OCH_2_*Me*) and 0.88 (3H, t, *J* = 6.5, C_4_H_8_*Me*); δ_C_ (50 MHz, CDCl_3_) 170.3, 169.5, 164.0, 82.9, 81.0, 64.1, 58.0, 54.2 (br), 39.2, 32.1, 31.6, 27.9, 25.9, 25.6, 22.8, 22.4, 18.9, 14.7 and 13.9; *m/z* (EI) 367 (3%, M^+^), 322 (5), 310 (3), 294 (3), 280 (5), 266 (20), 252 (16), 240 (11), 224 15), 223 (20), 182 (15), 170 (48), 169 (59), 168 (19), 150 (31), 149 (100), 124 (22), 104 (18), 100 (30), 98 (19), 97 (69), 96 (23), 82 (30) and 57 (100) (Found: M^+^, 367.2710. C_21_H_37_NO_4_ requires 367.2722).

**(3*R*)-3-{Benzyl[(1*R*)-1-phenylethyl]amino}-1-octanol 30**

A solution of *tert*-butyl (3*R*)-3-{benzyl[(1*R*)-1-phenylethyl]amino}octanoate (+)-**14** [1] (2.00 g, 4.88 mmol) in dry diethyl ether (20 cm^3^) was added dropwise with stirring to a suspension of LiAlH_4_ (0.20 g, 5.37 mmol) in dry diethyl ether (10 cm^3^) at 0 °C. The reaction mixture was stirred at room temperature for 2.75 h before being quenched by the sequential addition of H_2_O (0.20 cm^3^), aqueous NaOH solution (15% w/v, 0.20 cm^3^) and H_2_O (3 × 0.20 cm^3^). EtOAc (60 cm^3^) was added to the resulting white precipitate, and the mixture was stirred for 30 min before filtration through a pad of celite. The celite pad was washed with EtOAc, and the combined filtrates were dried (MgSO_4_), filtered and evaporated *in vacuo* to yield a crude yellow oil (1.70 g), which was purified by column chromatography on silica gel with hexane-EtOAc (85:15) as eluent to give *(3*R*)-3-{benzyl[(1*R*)-1-phenylethyl]amino}-1-octanol* **30** as a pale yellow oil (1.61 g, 97%); R*_f_* 0.35 (hexane-EtOAc 4:1); [α]_D_^23^ +8.1 (*c* 1.11, absolute EtOH); *v*_max_(film/cm^−1^) 3363 (m, v br, OH), 3084 (w), 3061 (w), 3027 (w), 2930 (s), 2858 (s), 1601 (w, C=C), 1493 (m), 1453 (s), 1374 (m), 1052 (m) and 1028 (m); δ_H_ (300 MHz, CDCl_3_) 7.39–7.19 (10H, m, Ar−H), 3.94 (1H, q, *J* = 6.9, PhC*H*N), 3.83 (1H, d, *J* = 13.8, NC*H*_a_H_b_Ph), 3.67 (1H, d, *J* = 13.8, NCH_a_*H*_b_Ph), 3.48 (1H, ddd, *J* = 10.8, 6.1 and 4.6, C*H*_a_H_b_OH), 3.20 (1H, ddd, *J* = 10.8, 7.4 and 4.2, CH_a_*H*_b_OH), 3.0–2.6 (1H, br s, O*H*), 2.79–2.74 (1H, m, C*H*N), 1.70–1.65 (1H, m, NCHC*H*_a_H_b_), 1.55–1.50 (1H, m, NCHCH_a_*H*_b_), 1.38 (3H, d, *J* = 6.9, PhCH*Me*), 1.35–1.19 (8H, m) and 0.91 (3H, t, *J* = 6.9, C_4_H_8_*Me*); δ_C_ (75 MHz, CDCl_3_) 143.8, 140.7, 129.0, 128.3, 128.1, 128.0, 126.9, 126.8, 61.8, 56.7, 55.2, 49.8, 33.6, 32.5, 32.1, 27.3, 22.6, 15.1 and 14.0; *m/z* (EI) 339 (5%, M^+^); 324 (5), 295 (14), 294 (56), 269 (18), 268 (86), 190 (48), 170 (11), 164 (73), 146 (5), 106 (14), 105 (100), 99 (23), 91 (81), 57(12) and 55 (12) (Found: M^+^, 339.2500. C_23_H_33_NO requires 339.2503).

**(3*R*)-*N*-Benzyl-1-{*tert*-butyl(dimethyl)silyl]oxy}-*N*-[(1*R*)-1-phenylethyl]-3-octanamine 31**

A solution of imidazole (1.71 g, 25.09 mmol) and (3*R*)-3-{benzyl[(1*R*)-1-phenylethyl]amino}-1-octanol **30** (4.26 g, 12.55 mmol) in dry DMF (15 cm^3^) was stirred at room temperature for 30 min prior to the addition of a solution of *tert*-butyl(dimethyl)silyl chloride (2.08 g, 13.80 mmol) in dry DMF (7 cm^3^). The resulting solution was stirred at room temperature under a nitrogen atmosphere for 18 h. The reaction mixture was diluted with ice and water (150 cm^3^), then extracted with CH_2_Cl_2_ (5 × 70 cm^3^). The combined extracts were dried (Na_2_SO_4_), filtered and evaporated *in vacuo*. The resulting residue was dissolved in CH_2_Cl_2_ (150 cm^3^) and washed several times with water (4 × 70 cm^3^). The organic phase was dried (Na_2_SO_4_), filtered and evaporated *in vacuo* to yield a crude pale yellow oil which was purified by column chromatography on silica gel with hexane-EtOAc (9:1) as eluent to give *(3*R*)-*N*-benzyl-1-{tert-butyl(dimethyl)silyl]oxy}-*N*-[(1*R*)-1-phenylethyl]-3-octanamine* **31** as a colourless oil (5.62 g, 99%); R*_f_* 0.89 (hexane-EtOAc 4:1); [α]_D_^21^ −7.3 (*c* 1.17, absolute EtOH); *v*_max_(film/cm^−1^) 3028 (w), 2956 (s), 2930 (s), 2857 (s), 1493 (w), 1462 (m), 1254 (m), 1094 (s), 909 (m) and 836 (s); δ_H_ (300 MHz, CDCl_3_) 7.42–7.19 (10H, m, Ar−H), 3.89 (1H, q, *J* = 6.9, PhC*H*N), 3.79 (1H, d, *J* = 14.9, NC*H*_a_H_b_Ph), 3.66 (1H, d, *J* = 14.9, NCH_a_*H*_b_Ph), 3.48 (1H, ddd, *J* = 10.0, 8.0 and 6.7, CH_a_H_b_OSi), 3.29 (1H, ddd, *J* = 9.9, 8.6 and 5.4, CH_a_*H*_b_OSi), 2.68 (1H, quintet, *J* = 6.2, C*H*N), 1.62–1.55 (1H, m), 1.54–1.45 (2H, m), 1.37–1.18 and 1.31 (10H, overlapping m and d, *J* = 7.0, alkyl-*H* and PhCH*Me*), 0.90 and 0.88 (12H, overlapping t and s, *J* = 7.3, C_4_H_8_*Me* and SiC*Me*_3_) and 0.004 (6H, s, Si*M*e_2_); δ_C_ (75 MHz, CDCl_3_) 144.9, 142.7, 128.3, 128.1, 128.0, 127.9, 126.5, 126.4, 62.0, 58.1, 54.0, 50.2, 34.6, 32.9, 32.1, 27.1, 26.0, 22.7, 18.8, 18.3, 14.1 and –0.5; *m/z* (EI) 453 (2%, M^+^), 384 (8), 383 (32), 382 (100), 295 (19), 294 (80), 190 (42), 146 (8), 106 (10), 105 (87), 91 (49), 89 (11) and 73 (15) (Found: M^+^, 453.3435. C_29_H_47_NOSi requires 453.3427).

**(3*R*)-1-{[*tert*-Butyl(dimethyl)silyl]oxy}-3-octanamine 32**

Palladium hydroxide on activated carbon (20%; 1.16 g) was suspended in a solution of protected alcohol **31** (3.51 g, 7.74 mmol) in absolute EtOH (70 cm^3^). The mixture was stirred in an autoclave at room temperature under hydrogen gas (5 atm) for 16 h. The catalyst was filtered off through a pad of celite, which was washed with EtOH. The filtrate was evaporated *in vacuo* to afford a dark yellow oil (2.29 g), which oil was purified by column chromatography on silica gel with EtOAc as eluent to give *(3*R*)-1-{[*tert*-butyl(dimethyl)silyl]oxy}-3-octanamine* **32** as a yellow oil (2.00 g, 100%); R*_f_* 0.11 (MeOH-EtOAc 1:9); [α]_D_^22^ −7.5 (*c* 1.33, absolute EtOH); *v*_max_(film/cm^−1^) 3368 (m, br, N−H), 2956 (s), 2932 (s), 2859 (m), 1586 (m), 1457 (s), 1382 (m), 1207 (m), 1063 (s), 766 (s) and 703 (s); δ_H_ (300 MHz, CDCl_3_) 3.70–3.65 (2H, m, C*H*_2_OSi), 2.81–2.79 (1H, m, C*H*N), 1.61–1.56 (1H, m, NCHC*H*_a_H_b_), 1.42–1.35 (5H, m, NH_2_, NCHCH_a_*H*_b_, C*H*_2_), 1.34–1.22 (6H, m), 0.85–0.82 (12H, m, C_4_H_8_*Me* and SiC*Me*_3_) and 0.01 (6H, s, Si*M*e_2_); δ_C_ (75 MHz, CDCl_3_) 61.1, 49.0, 40.5, 38.4, 31.9, 25.9, 25.7, 22.6, 18.2, 14.0 and –1.0; *m/z* (EI) 244 (5%, M^+^ – Me), 202 (26), 188 (18), 170 (8), 116 (5), 104 (13), 100 (66), 89 (16), 75 (24), 74 (100),73 (18) 69 (14), 56 (18) and 55 (17) (Found: M^+^ – Me, 244.2088. C_13_H_30_NOSi requires 244.2096).

**1-[(1*R*)-1-(2-{[*tert*-Butyl(dimethyl)silyl]oxy}ethylhexyl]-2-piperidinone 34**

5-Bromopentanoyl chloride (1.84 g, 9.25 mmol) was added to a stirred solution of primary amine **32** (2.00 g, 7.71 mmol) in dry 1,2-dichloroethane (30 cm^3^), followed by anhydrous NaHCO_3_ (0.78 g, 9.25 mmol). The reaction mixture was left to stir at room temperature for 16 h. The resulting mixture was filtered through a pad of celite, which was then washed with CH_2_Cl_2_. The combined filtrate and washings were then evaporated *in vacuo* to yield a crude orange oil (4.06 g), which was purified by column chromatography on silica gel with hexane-EtOAc (7:3) as eluent to give the *5-bromo-*N*-[(*1R*)-1-(2-{[*tert*-butyl(dimethyl)silyl]oxy}ethyl)hexyl pentanamide* **33** as an orange oil (2.92 g, 89%); R*_f_* 0.64 (hexane-EtOAc 7:3); δ_H_ (300 MHz, CDCl_3_) 6.07 (1H, br d, *J* 8.3, N*H*), 4.05–4.00 (1H, m, C*H*N), 3.80–3.75 (1H, m, C*H*_a_H_b_OSi), 3.69 (1H, ddd, *J* = 10.5, 5.7 and 4.8, CH_a_*H*_b_OSi), 3.41 (2H, t, *J* = 6.7, C*H*_2_Br), 2.15 (2H, t, *J* = 7.3, C*H*_2_C=O), 1.92–1.86 (2H, m), 1.82–1.76 (3H, m), 1.63–1.58 (1H, m), 1.52–1.45 (2H, m), 1.30–1.25 (4H, m), 0.91 and 0.88 (12H, overlapping s and t, *J* = 6.8, SiC*Me*_3_ and C_4_H_8_*Me*) and 0.07 (6H, s, Si*M*e_2_); δ_C_ (75 MHz, CDCl_3_) 171.3, 60.4, 47.6, 35.9, 35.8, 34.1, 33.1, 32.2, 25.9, 25.7, 24.3, 22.5, 18.1, 14.0 and –5.5 (Found: M^+^ – CH_3_, 406.1789. C_18_H_37_^79^BrNO_2_Si requires 406.1777). A portion of the bromoamide **33** (0.20 g, 0.47 mmol) was dissolved in dry THF (3 cm^3^) containing a suspension of potassium *tert*-butoxide (58 mg, 0.52 mmol), and the mixture was stirred at room temperature for 25 min before being diluted with EtOAc (10 cm^3^). The mixture was then washed with saturated aqueous sodium chloride solution (5 × 2 cm^3^). The combined organic extracts were dried (MgSO_4_), filtered and evaporated *in vacuo* to yield a crude yellow oil (0.16 g), which was purified by column chromatography on silica gel with hexane-EtOAc (85:15) as eluent to give *1-[(1*R*)-1-(2-{[*tert*-butyl(dimethyl)silyl]oxy}ethylhexyl]-2-piperidinone* **34** as a pale yellow oil (0.13 g, 81%); R*_f_* 0.22 (hexane-EtOAc 7:3); [α]_D_^24^ +8.5 (*c* 1.06, absolute EtOH); *v*_max_(film/cm^−1^) 2965 (s), 2929 (s), 2858 (s), 1645 (s, C=O), 1488 (w), 1463 (m), 1444 (m), 1417 (w), 1348 (w), 1329 w), 1254 (m), 1175 (m), 1095 (s), 837 (s) and 776 (m); δ_H_ (300 MHz, CDCl_3_) 4.75–4.55 (1H, br m, C*H*N), 3.60 (1H, ddd, *J* = 10.2, 8.2 and 6.7, C*H*_a_H_b_OSi), 3.50 (1H, ddd, *J* = 10.2, 8.4 and 5.5, CH_a_*H*_b_OSi), 3.12–3.06 (2H, m, C*H*_2_N), 2.37 (2H, br t, *J = ca* 6.3), 1.75–1.70 (5H, m), 1.70–1.63 (1H, m), 1.4—1.15 (2H, m), 1.27–1.15 (6H, m), 0.84 and 0.83 (12H, overlapping s and t, *J* = 7.1, SiC*M*e_3_ and C_4_H_8_*Me*); and 0.01 (6H, s, Si*M*e_2_); δ_C_ (75 MHz, CDCl_3_) 169.8, 60.9, 50.5 (br), 41.8 (br), 35.3, 32.4, 32.0, 31.6, 25.9, 25.8, 23.2, 22.5, 21.0, 18.2, 14.0 and −5.4; *m/z* (EI) 341 (0.5%, M^+^), 326 (3), 285 (22), 284 (100), 270 (3), 240 (4), 196 (6), 182 (25), 156 (48), 138 (11), 126 (16), 100 (18), 75 (7), 73 (13) and 55 (12) (Found: M^+^, 341.2738. C_19_H_39_O_2_NSi requires 341.2750).

**1-[(1*R*)-1-(2-Hydroxyethyl)hexyl]-2-piperidinone 35**

Hydrofluoric acid (40%; 5.50 cm^3^, 2.50 g, 0.13 mmol) was carefully added to a stirred solution of 1-[(1*R*)-1-(2-{[*tert*-butyl(dimethyl)silyl]oxy}ethylhexyl]-2-piperidinone **34** (1.18 g, 3.47 mmol) in MeOH (130 cm^3^). The reaction was stirred at room temperature for 100 min before being added to saturated aqueous NaHCO_3_ solution (200 cm^3^). The reaction mixture was then extracted with EtOAc (3 × 100 cm^3^). The combined extracts were dried (MgSO_4_), filtered and evaporated *in vacuo* to yield a pale yellow oil, which was purified by column chromatography on silica gel with EtOAc-hexane (4:1) as eluent to give *1-[(1*R*)-1-(2-hydroxyethyl)hexyl]-2-piperidinone* **35** as a pale yellow oil (0.70 g, 89%); R*_f_* 0.13 (80% EtOAc-hexane); [α]_D_^24^ +40.8 (*c* 1.20, absolute EtOH); *v*_max_(film/cm^−1^) 3410 (v br, OH), 2965 (s), 2930 (s), 2860 (s), 1616 (s, C=O), 1492 (m), 1467 (m), 1418 (m), 1350 (m), 1331 (m), 1176 (m) and 1055 (m); δ_H_ (300 MHz, CDCl_3_) 4.80–4.70 (1H, m, C*H*N), 3.8–3.5 and 3.55 (2H, overlapping br s and ddd, *J* = 11.9, 5.8 and 2.8, OH and C*H*_a_H_b_OH), 3.33 (1H, td, *J* = 11.6 and 3.1, CH_a_*H*_b_OH), 3.14–3.08 (1H, m, NC*H*_a_H_b_), 3.04–2.98 (1H, m, NCH_a_*H*_b_), 2.50 and 2.43 (2H, 2 ×dt, *J* = 18.1 and 6.7, C*H*_a_*H*_b_CON), 1.88–1.67 (4H, m), 1.59–1.39 (4H, m), 1.30–1.23 (6H, m) and 0.88 (3H, t, *J* = 6.8, C_4_H_8_*Me*); δ_C_ (75 MHz, CDCl_3_) 171.7, 58.2, 48.8, 40.5, 34.0, 32.0, 31.6, 31.4, 25.9, 22.8, 22.3, 20.6 and 13.8; *m/z* (EI) 227 (15%, M^+^), 196 (23), 183 (15), 182 (94), 170 (10), 157 (12), 156 (100), 138 (35), 126 (64), 112 (13), 100 (58), 82 (11) and 55 (20) (Found: M^+^, 227.1879. C_13_H_25_NO_2_ requires 227.1885).

**(3*R*)-3-(2-Oxo-1-piperidinyl)octyl acetate 36**

A solution of acetic anhydride (0.39 cm^3^, 0.42 g, 4.16 mmol) in dry pyridine (0.30 cm^3^, 0.35 g, 4.16 mmol) was added dropwise to a solution of alcohol **35** (0.63 g, 2.77 mmol) in dry pyridine (0.24 cm^3^, 0.23 g, 2.77 mmol) at 0 °C. The reaction was left to stir at room temperature for 16 h. EtOAc (15 cm^3^) was added, and the mixture was washed with saturated aqueous NaCl solution (3 × 20 cm^3^). The combined aqueous washings were further extracted with CH_2_Cl_2_ (3 × 20 cm^3^). The combined organic extracts were washed with saturated aqueous NH_4_Cl solution containing concentrated aqueous NH_4_OH solution (5 cm^3^). The organic phase was dried (MgSO_4_), filtered and evaporated *in vacuo* to yield a crude yellow oil (0.84 g), which was purified by column chromatography on silica gel with EtOAc-hexane (1:1) as eluent to give *(3*R*)-3-(2-oxo-1-piperidinyl)octyl acetate* **36** as a pale yellow oil (0.75 g, 100%); R*_f_* 0.51 (EtOAc-hexane 1:1); [α]_D_^24^ +13.2 (*c* 1.66, absolute EtOH); *v*_max_(film/cm^−1^) 2965 (s), 2931 (s), 2860 (s), 1738 (s, C=O), 1638 (s, br, C=O), 1490 (m), 1466 (m), 1418 (m), 1368 (m), 1330 (m), 1263 (s), 1176 (m) and 1036 (m); δ_H_ (300 MHz, CDCl_3_) 4.75–4.73 (1H, br s, C*H*N), 4.04–4.00 (2H, m, C*H*_2_OAc), 3.12–3.08 (2H, m, NC*H*_2_), 2.41 (2H, t, *J* = 6.5, C*H*_2_CON), 2.04 (3H, s, CO*Me*), 1.83–1.74 (6H, m), 1.51–1.41 (2H, m, NCHC*H*_2_Bu), 1.30–1.20 (6H, m) and 0.87 (3H, t, *J* = 6.9, C_4_H_8_*Me*); δ_C_ (75 MHz, CDCl_3_) 171.1, 170.3, 61.6, 49.5 (br), 41.1 (br), 32.3, 31.8, 31.6, 30.7, 25.7, 23.1, 22.4, 20.9, 20.8 and 13.9; *m/z* (EI) 269 (10%, M^+^), 210 (5), 196 (8), 182 (41), 152 (10), 139 (14), 138 (100), 152 (10), 100 (23) and 55 (10) (Found: M^+^, 269.2006. C_15_H_27_NO_3_ requires 269.1991).

**(3*R*)-3-(2-Thioxo-1-piperidinyl)octyl acetate 37**

Lawesson’s reagent (0.56g, 1.39 mmol, 0.5 equiv.) was added to a stirred solution of (3*R*)-3-(2-oxo-1-piperidinyl)octyl acetate **36** (0.75 g, 2.78 mmol) in dry toluene (10 cm^3^). The mixture was heated under reflux for 135 min before the solvent was evaporated *in vacuo* to yield an orange oil. Purification by column chromatography on silica gel with CH_2_Cl_2_-hexane (4:1) as eluent yielded *(3*R*)-3-(2-thioxo-1-piperidinyl)octyl acetate* **37** as a yellow oil (0.74 g, 94%); R*_f_* 0.33 (hexane-EtOAc 7:3); [α]_D_^20^ +6.3 (*c* 1.11, absolute EtOH); *v*_max_(film/cm^−1^) 2965 (s), 2932 (s), 2860 (s), 1734 (s, C=O), 1497 (s), 1443 (s), 1350 (m, br, C=S), 1236 (s), 1137 (m), 1106 (m) and 1039 (m); δ_H_ (300 MHz, CDCl_3_) 6.07 (1H, br quintet, *J* = *ca* 6.0, C*H*N), 4.13–4.02 (2H, m, C*H*_2_OAc), 3.33–3.21 (2H, m, NC*H*_2_), 3.03 (2H, td, *J* = 6.7 and 1.4, C*H*_2_C=S), 2.05 (3H, s, CO*Me*), 1.93–1.83 (4H, m), 1.78–1.70 (2H, m), 1.59–1.58 (2H, m, NCHC*H*_2_Bu), 1.20–1.40 (6H, m) and 0.88 (3H, t, *J* = 7.0, C_4_H_8_*Me*); δ_C_ (75 MHz, CDCl_3_) 202.0, 170.8, 61.2, 56.9, 42.7, 42.0, 32.2, 31.6, 31.3, 25.4, 22.2 (two overlapping signals), 20.9, 19.6 and 13.8; *m/z* (EI) 286 (11), 285 (56%, M^+^), 284 (35), 253 (17), 252 (100), 226 (11), 212 (10), 192 (10), 182 (11), 168 (10), 166 (16), 154 (13), 138 (18), 129 (15), 116 (86), 115 (47), 114 (20), 82 (38), 67 (11) and 55 (30) (Found: M^+^, 285.1761.C_15_H_27_NO_2_S requires 285.1763).

**Ethyl (2*E*)-(1-{(1*R*)-[2-(acetyloxy)ethyl]hexyl}-2-piperidinylidene)ethanoate 38**

Ethyl bromoacetate (0.20 cm^3^, 0.30 g, 1.77 mmol) was added to a stirred solution of thiolactam **37** (0.46 g, 1.61 mmol) in dry MeCN (3 cm^3^). The solution was left to stir at room temperature under a nitrogen atmosphere for 16 h, at which stage TLC analysis showed that the salt formation was complete. The solvent and excess ethyl bromoacetate were evaporated *in vacuo*, and the residue was dissolved in a fresh portion of dry MeCN (3 cm^3^). This was followed by the addition of triphenylphosphine (0.46 g, 1.77 mmol) and triethylamine (0.25 cm^3^, 0.18 g, 1.77 mmol). On addition of the base, a milky peach-coloured solution was formed. The mixture was left to stir at room temperature under a nitrogen atmosphere for 16 h, after which water (15 cm^3^) was added. The solution was extracted with CH_2_Cl_2_ (3 × 30 cm^3^). The combined extracts were then dried (MgSO_4_), filtered and evaporated *in vacuo* to yield a crude orange oil (1.16 g), which was purified by column chromatography on silica gel with hexane-benzene (1:9) to remove phosphine residues, followed by hexane-EtOAc (4:1) hexane as eluent. *Ethyl (2*E*)-(1-{(1*R*)-[2-(acetyloxy)ethyl]hexyl}-2-piperidinylidene)ethanoate* **38** was obtained as a pale yellow oil (0.44 g, 80%); R*_f_* 0.83 (hexane-EtOAc 1:1); [α]_D_^20^ +32.6 (*c* 1.18, absolute EtOH); *v*_max_(film/cm^−1^) 2960 (m), 2932 (m), 2861 (m), 1741 (s, C=O), 1681 (s, C=C), 1568 (s), 1464 (w), 1423 (w), 1381 (w), 1367 (w), 1239 (s), 1146 (s), 1075 (m), 1049 (m) and 791 (m); δ_H_ (300 MHz, CDCl_3_) 4.78 (1H, s, *H*C=), 4.07–3.97 (5H, m, C*H*N, C*H*_2_OAc and OC*H*_2_Me), 3.21 and 3.13 (2H, 2 × dt, *J* = 10.5 and 6.7, NC*H*_a_*H*_b_), 3.06–3.02 (2H, m, C*H*_2_C=), 2.08 (3H, s, CO*Me*), 1.81–1.63 (6H, m), 1.60–1.40 (2H, m, NCHC*H*_2_Bu), 1.35–1.19 and 1.23 (9H, overlapping m and t, *J* = 7.1, 3 × CH_2_ and OCH_2_*Me*) and 0.88 (3H, t, *J* = 6.8, C_4_H_8_*Me*); δ_C_ (75 MHz, CDCl_3_) 171.0, 169.5, 164.8, 81.9, 61.3, 58.0, 53.4, 39.5, 32.3, 31.7, 31.2, 25.9, 25.5, 22.8, 22.4, 20.8, 18.9, 14.6 and 13.9; *m/z* (EI) 339 (27%, M^+^), 338 (15), 294 (59), 280 (18), 266 (29), 253 (16), 170 (59), 252 (90), 208 (40), 169 (100), 124 (28), 97 (59), 82 (23), 69 (21) and 55 (30) (Found: M^+^, 339.2419. C_19_H_33_O_4_N requires 339.2410).

**Ethyl (2*E*)-{1-[(1*R*)-1-(2-hydroxyethyl)hexyl]-2-piperidinylidene}ethanoate 29**

(a) LiAlH_4_ (45.5 mg, 1.30 mmol) was added to a stirred solution of *tert-*butyl (3*R*)-3-[(2*E*)-2-(2-ethoxy-2-oxoethylidene)piperidinyl]octanoate **28** (0.21 g, 0.57 mmol) in dry THF (10 cm^3^). The mixture was stirred at room temperature for 16 h, after which a further portion of the hydride was added. After another 3 h, TLC analysis showed no sign of further reaction. The reaction was quenched by the sequential addition of H_2_O (0.20 cm^3^), aq. NaOH solution (15% w/v; 0.20 cm^3^) and H_2_O (3 × 0.20 cm^3^). The white precipitate was filtered off through a pad of celite, which was washed with CH_2_Cl_2_. The combined filtrates were dried (MgSO_4_), filtered and evaporated *in vacuo* to yield a yellow oil (0.15 g), which was purified by column chromatography as described above to give recovered starting material (20 mg, 10%) and *ethyl (2*E*)-{1-[(1*R*)-1-(2-hydroxyethyl)hexyl]-2-piperidinylidene}ethanoate* **29** as a pale yellow oil (49 mg, 29%); characterisation as described below.

(b) Potassium carbonate (0.20 g, 1.46 mmol) was added to a stirred solution of ethyl (2*E*)-(1-{(1*R*)-[2-(acetyloxy)ethyl]hexyl}-2-piperidinylidene)ethanoate **38** (0.33 g, 0.97 mmol) in MeOH (2 cm^3^). The solution was stirred for 2.75 h before being filtered through a pad of celite. The celite was washed with MeOH, and the combined filtrates were evaporated *in vacuo* to yield a crude oil (0.48 g), which was purified by column chromatography on silica gel with hexane-EtOAc-hexane (4:1) as eluent to give e*thyl (2*E*)-{1-[(1*R*)-1-(2-hydroxyethyl)hexyl]-2-piperidinylidene}ethanoate* **29** as an orange oil (0.26 g, 70%); R*_f_* 0.58 (hexane-EtOAc 1:1); [α]_D_^18^ +13.9 (*c* 1.01, absolute EtOH); *v*_max_(film/cm^−1^) 3410 (v br, OH), 2934 (s), 2861 (s), 1729 (m, C=O), 1677 (s, C=C), 1446 (m), 1380 (w), 1144 (s), 1072 (m) and 790 (w); δ_H_ (300 MHz, CDCl_3_) 4.81 (1H, s, *H*C=), 4.13 (2H, q, *J* = 7.1, OC*H*_2_Me), 3.99–3.82 (1H, m, C*H*N), 3.66–3.55 (2H, m, C*H*_2_OH), 3.18 (2H, t, *J* = 6.5, NC*H*_2_), 3.15–3.13 (2H, m, C*H*_2_C=), 1.75–1.60 (6H, m), 1.59–1.43 (2H, m, NCHC*H*_2_Bu), 1.27–1.21 and 1.26 (9H, overlapping m and t, *J* = 7.1, 3 × CH_2_ and OCH_2_*Me*) and 0.88 (3H, t, *J* = 6.8, C_4_H_8_*Me*); δ_C_ (75 MHz, CDCl_3_) 171.8, 169.7, 81.2, 61.0, 59.7, 58.1, 36.3, 35.6, 32.4, 32.0, 31.7, 26.1, 25.6, 22.5, 18.8, 14.6 and 13.9; *m/z* (EI) 297 (6%, M^+^), 294 (44), 280 (14), 266 (28) 252 (84), 226 (14), 222 (17), 210 (83), 208 (27), 182 (18), 170 (61), 169 (100). The compound was difficult to purify, and was used as obtained in the following reaction.

**Ethyl (4*R*)-4-pentyl-3,4,6,7,8,9-hexahydro-2*H*-quinolizine-1-carboxylate 39**

The alcohol **29** (284 mg, 0.95 mmol), triphenylphosphine (0.75 g, 2.86 mmol) and imidazole (0.19 g, 2.86 mmol) were dissolved in a mixture of toluene (3 cm^3^) and MeCN (6 cm^3^). Once the solids had dissolved, iodine (0.48 g, 1.91 mmol) was added. The homogeneous solution was then stirred under reflux for 45 min. The reaction was quenched by the addition of saturated aqueous NaHCO_3_ solution (20 cm^3^), after which the mixture was extracted with EtOAc (3 × 20 cm^3^). The combined organic extracts were washed with saturated aqueous Na_2_S_2_O_3_ solution (20 cm^3^), then dried (MgSO_4_), filtered and evaporated *in vacuo* to yield an orange oil (1.22 g). The crude oil was purified by column chromatography on silica gel with CH_2_Cl_2_ as eluent to give *ethyl (4R)-4-pentyl-3,4,6,7,8,9-hexahydro-2H-quinolizine-1-carboxylate* **39** as a yellow oil (186 mg, 70%); R*_f_* 0.58 (50% EtOAc-hexane); [α]_D_^20^ –162.6 (*c* 1.03, absolute EtOH); *v*_max_(film/cm^−1^) 2932 (s), 2858 (m), 1732 (m, C=O), 1671 (m, C=C), 1550 (m), 1463 (m), 1263 (m), 1193 (s), 1100 (m) and 1070 (m); δ_H_ (300 MHz, CDCl_3_) 4.1–4.04 (2H, overlapping q, *J* = *ca* 7.1, OC*H_a_H*_b_Me), 3.26 (1H, dt, *J* = 12.0 and 6.0, 6a-H), 3.17 (1H, dt, *J* = 12.3 and 6.1, 6b-H), 3.20–3.09 (1H, m, 4-H), 3.00–2.86 (2H, 2 × m, 9-H), 2.50 (1H, dd, *J* = 16.4 and 4.9, 3a-H), 2.23–2.14 (1H, m, 3b-H), 1.79–1.45 (8H, m), 1.25 (3H, t, *J* = 7.1, OCH_2_*Me*), 1.39–1.21 (6H, m), 0.89 (3H, t, *J* = 6.8, C_4_H_8_*Me*); δ_C_ (75 MHz, CDCl_3_) 169.0, 155.5, 90.5, 59.4, 58.3, 50.2, 31.8, 30.6, 27.7, 25.7, 23.6, 23.4, 22.6, 20.3, 19.5, 14.7 and 14.0; *m/z* (EI) 279 (53%, M^+^), 250 (23), 236 (21), 234 (66), 222 17), 209 (80), 208 (100), 206 (69), 194 (10), 180 (18), 136 (73), 134 (35) and 55 (11) (Found: M^+^, 279.2193. C_17_H_29_NO_2_ requires 279.2198).

**Ethyl (1*S*,4*R*,9a*S*)-4-pentyloctahydro-2*H*-quinolizine-1-carboxylate 40**

Platinum dioxide catalyst (29 mg) was added to a solution of ethyl (4*R*)-4-pentyl-3,4,6,7,8,9-hexahydro-2*H*-quinolizine-1-carboxylate **39** (0.16 g, 0.59 mmol) in absolute EtOH (8 cm^3^) in an autoclave. The mixture was stirred under hydrogen (5 atm) at room temperature for 16 h. The solution was then filtered through a pad of celite, which was washed with EtOH. The combined filtrates were evaporated *in vacuo* to yield an orange oil (0.15 g), which was purified by column chromatography on silica gel with MeOH-EtOAc (1:9) as eluent to give, as the only observed diastereomer, *ethyl (1*S*,4*R*,9a*S*)-4-pentyloctahydro-2*H*-quinolizine-1-carboxylate* **40** as a yellow oil (0.16 g, 97%); R*_f_* 0.17 (MeOH-EtOAc 1:9); [α]_D_^21^ –24.6 (*c* 1.02, absolute EtOH); *v*_max_(film/cm^−1^) 2933 (s), 2859 (m), 2790 and 2753 (w, Bohlmann bands), 1741 (s, C=O), 1446 (m), 1378 (m), 1272 (m), 1153 (s), 1106 (m) and 1037 (m); δ_H_ (300 MHz, CDCl_3_) 4.17–4.08 (2H, 2 × overlapping q, *J* = 7.1, OC*H_a_H*_b_Me), 3.35–3.33 (1H, m), 2.49–2.46 (1H, m, H–1), 2.01–1.88 (3H, m,), 1.80–1.47 (11H, m), 1.42–1.31 (2H, m), 1.30–1.22 and 1.25 (10H, overlapping m and t, *J* = 7.1, C*H*_2_ and OCH_2_*Me*) and 0.88 (3H, t, *J* = 7.0, C_4_H_8_*Me*); δ_C_ (75 MHz, CDCl_3_) 173.5, 64.2, 64.0, 59.6, 52.6, 44.9, 33.4, 32.4, 31.6, 27.4, 27.1, 26.0, 25.4, 24.9, 22.6, 14.3 and 14.1; *m/z* (EI) 282 (9, M^+^ + H), 281 (3%, M^+^), 280 (4), 254 (12), 236 (20), 211 (42), 210 (100), 196 (20), 182 (26), 166 (13) and 136 (13) (Found: M^+^, 281.2342. C_17_H_31_NO_2_ requires 281.2355).

**(1*S*,4*R*,9a*S*)-4-Pentyloctahydro-2*H*-quinolizin-1-yl]methanol 41**

A solution of quinolizidine **40 (**0.16 g, 0.57 mmol) in dry diethyl ether (3 cm^3^) was added to a stirred suspension of LiAlH_4_ (32 mg, 0.85 mmol) in dry diethyl ether (1.5 cm^3^) at 0 °C. Stirring was continued for 1 h at this temperature and for a further 1 h at room temperature. The reaction mixture was then filtered through a pad of celite, which was washed with undried diethyl ether. The combined filtrate was evaporated *in vacuo* to yield a crude oil (0.21 g), which was purified by column chromatography on silica gel with EtOAc as eluent to give *(1*S*,4*R*,9a*S*)-4-pentyloctahydro-2*H*-quinolizin-1-yl]methanol* **41** as a pale yellow oil (88 mg, 65%); R*_f_* 0.25 (EtOAc-MeOH 4:1); [α]_D_^21^ –47.7 (*c* 1.09, absolute EtOH); *v*_max_(film/cm^−1^) 3351 (m, v br, OH), 2932 (s), 2861 (m), 2797 and 2764 (m, Bohlmann bands), 1447 (m), 1378 (m), 1349 (w), 1271 (w), 1093 (m), 1050 (s) and 881 (w); δ_H_ (300 MHz, CDCl_3_) 5.20–4.95 (1H, br s, O*H*), 4.19 (1H, ddd, *J* = 10.8, 4.5 and 1.5, C*H*_a_H_b_OH), 3.68 (1H, d, *J* = 10.8, CH_a_*H*_b_OH), 3.24–3.21 (1H, m), 2.23–2.17 (1H, m), 2.12–1.95 (2H, m), 1.87–1.73 (3H, m), 1.69–1.41 (10H, m), 1.40–1.18 (6H, m) and 0.89 (3H, t, *J* = 7.0, C_4_H_8_*Me*); δ_C_ (75 MHz, CDCl_3_) 65.8, 65.2, 64.0, 52.3, 38.4, 33.2, 32.4, 31.4, 30.7, 28.4, 26.1, 24.6, 23.5, 22.5 and 14.0; *m/z* (EI) 239 (1%, M^+^), 238 (2), 208 (2), 169 (11), 168 (100), 138 (4), 110 (3), 84 (6) and 55 (3) (Found: M^+^, 239.2228. C_15_H_29_NO requires 239.2249).

**References**

1. Michael JP, Gravestock D: *J Chem Soc, Perkin Trans 1* 2000:1919–1928.

2. Holmes AB, Smith AL, Williams SF, Hughes LR, Lidert Z, Swithenbank, C: *J Org Chem* 1991, **56:**1393–1405.

3. Covert LW, Adkins H: *J Am Chem Soc* 1932, **54:**4116–4117.

4. Collman JP, Groh, SE: *J Am Chem Soc* 1982; **104:**1391-1403.
